# Effect of Apigenin-Enriched *Elsholtzia splendens* Flower Extract on Lipid and Reactive Oxygen Species (ROS) Accumulation in 3T3-L1 Cells and *Caenorhabditis elegans*

**DOI:** 10.17113/ftb.63.04.25.8666

**Published:** 2025-12-26

**Authors:** Seulbi Lee, Soo-Im Choi, Miran Jang

**Affiliations:** 1 Department of Smart Food & drug, Inje University, 197, Inje-ro, Gimhae-si, Gyeongsangnam-do, Republic of Korea; 2 MEDIOGEN Co., Ltd. R&D Center, 120, Bio Valley 1-ro, Jecheon-si, Chungcheongbuk-do, Republic of Korea; 3 Department of Food Technology and Nutrition, Inje University, 197, Inje-ro, Gimhae-si, Gyeongsangnam-do, Republic of Korea

**Keywords:** *Elsholtzia splendens*, apigenin, lipid accumulation, reactive oxygen species (ROS), 3T3-L1 cells, *Caenorhabditis elegans*

## Abstract

**Research background:**

In the case of obesity, enlarged adipocytes cause an imbalance in lipid metabolism and increased oxidative stress, leading to excessive production of reactive oxygen species (ROS). ROS contribute to metabolic disorders such as inflammation and insulin resistance, further worsening lipid imbalance and promoting obesity-related diseases. Therefore, we investigated the benefits of *Elsholtzia splendens*, which simultaneously suppresses lipid accumulation and ROS.

**Experimental approach:**

We determined the total flavonoid content of the extracts and identified the functional ingredients by HPLC. Cell viability and proliferation were evaluated in 3T3-L1 cells. The total lipid and triglyceride (TG) contents in 3T3-L1 cells and the nematode *Caenorhabditis elegans* were measured using Oil Red O staining and TG assay, respectively. A quantitative polymerase chain reaction (qPCR) analysis was performed to investigate the mRNA quantity and accumulated ROS in nematodes.

**Results and conclusions:**

Apigenin was the major compound in the *E. splendens* extracts and was most abundant in the flower. The *E. splendens* flower extract did not show toxicity at concentrations of 25–100 μg/mL and had the highest apigenin content, thus we used the flower extract in the subsequent tests. *E. splendens* flower extract and apigenin inhibited total lipid and TG accumulation in 3T3-L1 cells and nematodes. These effects were attributed to the inhibition of peroxisome proliferator-activated receptor gamma (PPARγ) and CCAAT/enhancer‐binding protein α (C/EBPα) expression, which are involved in adipocyte differentiation. In addition, the flower extract and apigenin induced the nuclear localization of DAF-16, which is involved in lipogenesis in nematodes. The flower extract and apigenin also inhibited ROS accumulation in nematodes.

**Novelty and scientific contribution:**

Research on *E. splendens* has mainly focused on its cultivation and growth. Investigation of the effects of *E. splendens* on metabolic diseases, including obesity, has been limited and this study provides new insights. Our results suggest that *E. splendens* flower extract is a valuable material to inhibit lipid and ROS accumulation, and indicate that apigenin-rich functional plant materials should be considered as potential agents against obesity and related diseases.

## INTRODUCTION

Obesity is defined as an increase in body size and mass due to excessive accumulation of lipids, which not only affects body shape but also leads to chronic metabolic diseases such as type 2 diabetes, hyperlipidaemia, arteriosclerosis and cardiovascular disease (*1*). Therefore, the seriousness of obesity is indisputable. Among various therapeutic approaches, the most effective involves the suppression of fat accumulation in the body (*1*, *2*). Many gene products, including fatty acid synthase, fatty acid-binding protein, glucose transporter 4 and apolipoprotein, are involved in fat deposition during adipogenesis (*3*, *4*). Notably, peroxisome proliferator-activated receptor γ (PPARγ) contributes to the maturation of adipocytes and lipid accumulation in cooperation with CCAAT/enhancer‐binding protein α (C/EBPα) (*4*). Research on substances that suppress lipid accumulation by regulating the expressions of PPARγ and C/EBPα is ongoing (*5*).

Studies using *Caenorhabditis elegans* are conducted *via* high-throughput screening (HTS), which can bridge the gap between *in vitro* and *in vivo* mammalian model studies (*6*, *7*). DAF-16 is the only homologue of the forkhead box O (FOXO) family of transcription factors in *Caenorhabditis elegans* (*8*). DAF-16/FOXO is associated with insulin resistance and participates in apoptosis, cell cycle regulation, stress resistance and energy metabolism (*9*, *10*). In particular, FOXO1 is well known to be involved in the regulation of the cell cycle during differentiation (*9*-*11*). Many studies have shown that PPARγ is essential for adipocyte differentiation and that activated FOXO1 regulates PPARγ to inhibit adipogenesis (*9*-*11*). In addition, DAF-16/FOXO is closely related to resistance to oxidative stress and thus influences reactive oxygen species (ROS) generation (*12*).

Metabolic disorders with obesity commonly cause excessive ROS accumulation and can be fatal (*2*). Various studies have shown that imbalanced lipid metabolism in obesity causes metabolic stress and increases ROS production in the body (*13*). In particular, high-sugar diets accelerate oxidative stress, which is defined as glucose toxicity with hyperglycaemia (*14*). Therefore, to prevent chronic metabolic disorders, it is necessary to identify a substance with significant antioxidant activity that suppresses lipid accumulation (*15*, *16*).

*Elsholtzia splendens* is a perennial herb in the Lamiaceae family and has been used in traditional medicine. It is used as an ingredient in folk medicine to treat coughs, pain and inflammation in North-East Asia (*17*). The plant is aromatic and contains essential oils and compounds such as elsholtzia ketone, naginata ketone, isobutyl isovalerate, geraniol and geranyl acetate (*18*, *19*). Its phenolic contents include hesperidin, rutin, luteolin, quercetin and apigenin (*20*-*22*). Furthermore, the phenolics in *E. splendens* extracts have been reported to have positive effects on ageing through ROS inhibition, regulate microbial and viral growth, improve blood lipid levels, reduce inflammation and have beneficial effects on Alzheimer's disease (*20*, *22*-*24*). In particular, a previous study reported that *E. splendens* plants collected from various geographical regions commonly contain apigenin (*21*). Various plants containing apigenin and apigenin glycosides have antitumour and hepatoprotective effects and positively influence the cardiovascular and nervous systems (*25*-*27*). In addition, many studies have reported that apigenin regulates lipid metabolism (*26*-*28*).

This study investigated the total flavonoid and apigenin content in four parts of the plant (root, stem, leaf and flower). Total lipid and triglyceride (TG) contents were measured in 3T3-L1 cells and *C. elegans* treated with *E. splendens* flower extract (hereafter referred to as the flower extract), which has the highest flavonoid and apigenin content, to evaluate the inhibitory effect of the flower extract on lipid and ROS accumulation. We examined the molecular factors involved in fat deposition by analyzing PPARγ and C/EBPɑ in cells and DAF-16/FOXO in nematodes to understand the inhibitory effect of the flower extract on lipid accumulation.

## MATERIALS AND METHODS

### Reagents

Dulbecco's modified Eagle's medium (DMEM), foetal bovine serum (FBS), trypsin-EDTA solution, phosphate buffered saline (PBS), penicillin-streptomycin, and normal goat serum (NGS) were obtained from WellGENE Biopharmaceuticals (Daegu, Korea). RNA extraction kits (Trizol reagent) were purchased from Invitrogen Corp. (Carlsbad, CA, USA). The AccuPower^TM^ RT PreMix PCR kit, PPARγ, C/EBPɑ and glyceraldehyde-3-phosphate dehydrogenase (GAPDH) oligonucleotide primers were purchased from Bioneer Co. (Daejeon, Korea). All other chemicals were purchased from Sigma-Aldrich, Merck (St. Louis, MO, USA).

### Preparation of Elsholtzia splendens extracts

*Elsholtzia splendens* was collected during the flowering season from September to October in Jeollanam-do, Korea. The plants were completely dried, separated into flowers, leaves, roots and stems, and then powdered. The powders (10 g) were extracted with 80 % ethanol under reflux for 2 h. The extracts were then evaporated at 50 °C using a rotary evaporator (Eyela, Tokyo, Japan), lyophilized using a freeze-dryer (Ilshin, Seoul, Korea) to remove the remaining solvent, disolved in 0.1 % dimethyl sulfoxide (DMSO) and stored at 4 °C until required.

### Total flavonoid and apigenin contents

Total flavonoid and apigenin contents in *E. splendens* ethanolic extracts were analyzed as previously reported (*21*). HPLC analysis was performed using an UltiMate 3000 system (Thermo Scientific, Sunnyvale, CA, USA) equipped with a Capcell Pak C18VG 120 column (250 mm×4.6 mm, 5 µm). The mobile phase consisted of solvent A (0.1 % formic acid in water) and solvent B (0.1 % formic acid in acetonitrile). The gradient program was set from 80 to 45 % solvent A (20 to 55 % solvent B) for 15 min. The detection wavelength was 345 nm, with a flow rate of 1.0 mL/min and an injection volume of 10 µL. The concentration of apigenin contained in the extract was calculated using an apigenin calibration curve.

### Radical scavenging ability

To measure radical scavenging ability, a 2,2-diphenyl-1-picrylhydrazyl (DPPH) assay was performed (*21*). The sample and 0.2 mM DPPH solution were mixed in equal volumes and incubated for 30 min at room temperature. Trolox was used as a positive control. Absorbance was measured at 517 nm using a microplate reader (Biotek Synergy HTX; Agilent Technologies, Santa Clara, CA, USA) and radical scavenging activity (in %) was calculated as:


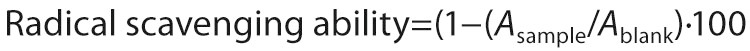
 /1/

### Evaluation of the in vitro effects of E. splendens extracts on adipocytes

#### Cell culture and adipocyte differentiation

3T3-L1 cells obtained from the American Type Culture Collection (ATCC) were seeded in 24-well plates (3·10^5^ cells/well). Two days after reaching confluence (day 1), adipogenic differentiation was induced using MDI (0.5 mM isobutylmethylxanthine (IBMX), 0.5 μM dexamethasone and 10 μg/mL insulin) prepared in DMEM supplemented with 10 % FBS. After the treatment with MDI, the cells were maintained in DMEM containing 10 μg/mL insulin until day 8, when differentiation was considered complete. The extracts (10–100 μg/mL) were added on day 0, 2 or 4 during differentiation and incubated until day 8. In this study, cells not exposed to MDI served as the non-differentiated (baseline) group, whereas cells treated with MDI served as the differentiated control group for comparison with extract-treated cells.

#### MTT assay

Cell viability was assessed using a 3-(4,5-dimethylthiazol-2-yl)-2,5-diphenyl-tetrazolium bromide (MTT) assay (*5*). Cells were seeded on 96-well plates at a density of 10^4^ cells per well, and 48 h after treatment, were incubated with 1 mg/mL MTT for 1 h. The MTT solution was then removed and the formazan produced was dissolved in DMSO. Cell viability was measured by spectrophotometry at 570 nm using a microplate reader (Biotek Synergy HTX; Agilent Technologies).

#### Cell proliferation assay

Cell proliferation was assessed by bromodeoxyuridine (BrdU)/propidium iodide (PI) immunofluorescence staining (*5*). Briefly, cells were seeded on glass coverslips in 3.5-cm dishes and differentiated as described. Cells were incubated with BrdU for 15 h and washed with PBS. They were then fixed and incubated with primary anti-BrdU monoclonal antibodies in 10 % NGS for 12 h at 4 °C, and then for 1 h with fluorescein isothiocyanate (FITC)-labelled secondary antibody and PI. Coverslips were washed with PBT, treated with PI solution (in PBS) for 30 min, mounted on slides, and observed under a fluorescence microscope (Eclipse Ci-L; Nikon, Seoul, Korea).

#### Oil Red O staining of adipocytes

3T3-L1 preadipocytes were induced to differentiate into mature adipocytes for 8 days, and then lipid accumulation was evaluated by Oil Red O (ORO) staining (*5*). Briefly, sample-treated cells were washed twice with PBS, fixed in 3.7 % paraformaldehyde, washed twice with 60 % isopropanol, and then stained with ORO solution for 1 h at room temperature. To quantify ORO uptake, cells were incubated with isopropanol, and the absorbance of the red-stained lipids was measured at 510 nm using a microplate reader (Biotek Synergy HTX; Agilent Technologies).

#### Triglyceride content of adipocytes

To analyze intracellular TG content, 3T3-L1 preadipocytes were washed twice with PBS and then scraped in 100 μL of lysis buffer (1 mM EDTA in 20 mM Tris). To measure TG content, lysates were reacted with TG assay reagent according to the manufacturer's instructions (Biomax, Guri-si, Gyeonggi-do, Korea) (*12*). Results are expressed as μg TG per μg cellular protein.

#### mRNA analysis

Total RNA was isolated from adipocytes with Trizol reagent and 1 μg was reverse-transcribed using RT Pre-mix. Quantitative polymerase chain reaction (PCR) was performed using an MJ Mini Gradient Thermal Cycler (Bio-Rad, Hercules, CA, USA). The used primer sequences are provided in Table S1. PCR amplification was carried out under the following conditions: an initial denaturation at 95 °C for 3 min, followed by 25 cycles of denaturation at 94 °C for 30 s, annealing at 55 °C for 30 s, and extension at 72 °C for 1 min. The final extension was performed at 72 °C for 5 min. PCR products were fractionated on 2 % agarose gels in 0.5× Tris–borate–EDTA (TBE) buffer and stained with ethidium bromide. Bands were visualized on a UV illuminator and recorded using an EL LoGic 100 Imaging System (Kodak, Tokyo, Japan). Images were analysed using Quantity One software (Bio-Rad). GAPDH was used as an internal control to calculate the abundance of mRNA in the sample. The band intensities were quantified using Image J (*22*).

### Evaluation of the in vivo effects of E. splendens flower extract using Caenorhabditis elegans

#### *C. elegans* cultivation

*C. elegans* (N2, wild type), TJ356 DAF-16::GFP (zls356) IV, and *Escherichia coli* OP50 were obtained from the Caenorhabditis Genetics Center (CGC, Minneapolis, MN, USA). Worms were cultured on nematode growth medium (NGM) plates at 20 °C. NGM plates were freshly prepared in our laboratory according to the standard protocol described in WormBook (*29*). Briefly, NaCl (3 g), agar (17 g) and peptone (2.5 g) were dissolved in distilled water (final volume 975 mL) and autoclaved. Then, 1 M CaCl_2_ (1 mL), 1 M MgSO_4_ (1 mL), cholesterol (1 mL, *γ*=5 mg/mL in ethanol) and 1 M potassium phosphate buffer (25 mL) were added. Bodies of mature worms were dissolved in 6 % household bleach, and the residues were washed multiple times with M9 buffer to collect embryos for the preparation of synchronized worms. To evaluate the effect of the extract in a metabolic disease model of obesity, we fed the nematodes a 2 % glucose diet throughout their growth.

#### Oil Red O staining of *C. elegans*

To quantify lipid accumulation in *C. elegans*, ORO staining was carried out (*8*). L4 stage worms were treated with *E. splendens* flower extract (25, 50 or 100 µg/mL) or apigenin (10, 20 or 40 µM) for 12 h. Young adult worms were grown in liquid media containing the flower extract or apigenin, washed three times with PBS, fixed with 3.7 % paraformaldehyde for 10 min and dehydrated with 60 % isopropanol for 5 min. Fixed worms were placed in ORO working solution for 2 h and washed with PBS. For imaging, approx. 20 worms were randomly selected under a Leica microscope equipped with DIC optics (Leica, Wetzlar, Germany) under identical settings and exposure times. ORO intensities were quantified using ImageJ (*22*).

#### Triglyceride contents of *C. elegans*

To quantify TG amount in worms, the worms were washed twice with PBS and dissolved in 500 μL PBS containing 0.05 % Tween 20, then homogenized on ice for 5 min. Homogenized samples were then centrifuged at 6000 ×*g* (Labogene 1730R; Gyrozen Co., Gimpo, Korea) for 5 min and supernatants were used for measurements. TG mass fractions were quantified using a free glycerol reagent, following the same kit and protocol as described for 3T3-L1 cells (*12*). Results were expressed as TG (μg) per cellular protein (mg).

#### Intensity of the reactive oxygen species in *C. elegans*

L2 worms were exposed to different concentrations of flower extract and apigenin for 50 h and then stained with 100 µM 2′,7′-dichlorodihydrofluorescein diacetate (H_2_DCF-DA) in the dark for 1 h. Worms were then mounted on slides with 2 % NaN_3_ and observed using a fluorescence microscope (Eclipse Ci-L; Nikon). Fluorescence intensity was analyzed using ImageJ (*22*), with approx. 15 worms per group used for quantification. This method was adapted, with modifications, from Kim *et al.* (*12*).

#### Nuclear translocation of DAF-16 in *C. elegans*

The DAF-16::GFP (zIs356) nematode is a transgenic mutant in which a green fluorescent protein is tagged to daf-16. We observed nuclear translocation of DAF-16 in adult nematodes after treatment with ESFE or apigenin for 50 h from L2 stage. Nematodes were fixed with 2 % NaN_3_ on slide glasses and observed under a fluorescence microscope (Eclipse Ci-L; Nikon). DAF-16::GFP expression patterns were classified as “cytoplasmic”, “intermediate” or “nuclear”, based on definitions from a previous study (*12*), to calculate the proportion of each group.

### Statistical analysis

Results were presented as the mean value±S.D. of three independent experiments. ANOVA or Student *t*-test in SPSS v. 19.0 (*23*) were used to determine the statistical differences. Significance was accepted for p<0.05 or p<0.01 as determined by the *t*-test.

## RESULTS AND DISCUSSION

### Total flavonoid and apigenin contents of Elsholtzia splendens extracts

In previous studies, the flavonoid content of *Elsholtzia splendens* varied greatly depending on its origin, cultivation environment, plant part and processing conditions (*19*, *30*, *31*). Therefore, qualitative and quantitative analyses of the active compounds needed to be prioritized before evaluating bioactivity. Previous studies have reported the presence of apigenin, quercetin and rutin in the flower extract (*19*), so we confirmed the presence of these compounds in this study.

**Fig. 1:**
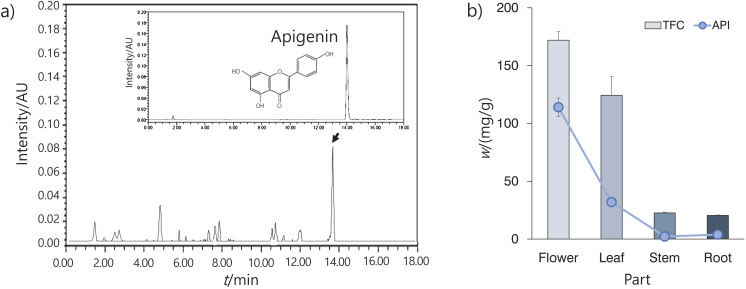
Total flavonoid (TFC) and apigenin (API) content on dry mass basis of different parts of *Elsholtzia splendens*: a) HPLC chromatogram of *E. splendens* flower extract and apigenin, and b) decrease of total flavonoid and apigenin mass fraction of different parts of *E. splendens*. Results are shown as the mean±S.D. of three independent experiments

To assess the potential of different parts of *E. splendens*, we examined the apigenin content in roots, stems, leaves and flowers. The total flavonoid and apigenin mass fractions of flowers, leaves, roots and stems are shown in Fig. 1. Apigenin was the predominant active compound in *E. splendens* (Fig. 1a). Both total flavonoid and apigenin mass fractions decreased in the order: flowers>leaves>roots>stems (Fig. 1b). The flowers contained the highest mass fractions of flavonoids and apigenin. In particular, apigenin was abundant in the flowers, accounting for 66.28 % of the total flavonoids. These results are similar to previous findings by Um and Kim (*21*).

### Effect of E. splendens extracts on 3T3-L1 cell viability

**Fig. 2:**
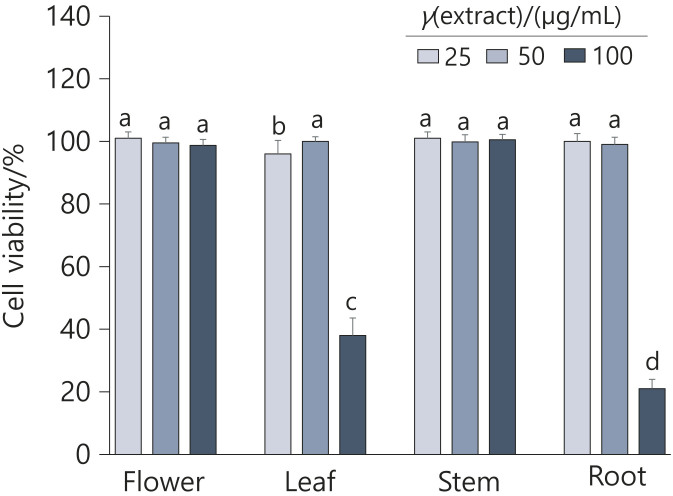
Effect of *Elsholtzia splendens* extracts on 3T3-L1 cell viability. 3T3-L1 preadipocytes were exposed to different parts of *E. splendens* extract (25, 50 and 100 µg/mL) for 48 h. Cell viability was measured using an MTT assay. Results are shown as the mean value±S.D. of three independent experiments. Different letters above the bars indicate significant differences at p<0.05

An MTT assay was used to examine the effects of *E. splendens* extracts on the viability of 3T3-L1 preadipocytes at extract concentrations of 25–100 μg/mL, corresponding to 17–68 μM of apigenin. No extract had a significant effect on the morphology or viability of confluent 3T3-L1 preadipocyte cells at concentrations of 25 or 50 μg/mL for 48 h. However, treatment with leaf and root extracts at 100 μg/mL significantly reduced cell viability (Fig. 2). The secondary metabolites present in different parts of a plant may vary, resulting in differing efficacy and toxicity profiles. These differences highlight the importance of considering the specific plant part used in the development of plant-based products (*32*). The flower extract was considered to have the greatest potential among the four parts due to its high apigenin content and lack of toxicity, and was used for further *in vitro* and *in vivo* investigations.

### Effects of the flower extract and apigenin on 3T3-L1 cell differentiation and proliferation during adipogenesis

Adipose tissue formation involves two main processes: the proliferation of preadipocytes and their differentiation into mature adipocytes (*33*). Recent studies have shown that obesity may be prevented by inducing adipocyte apoptosis and inhibiting adipogenesis (*34*, *35*).

**Fig. 3:**
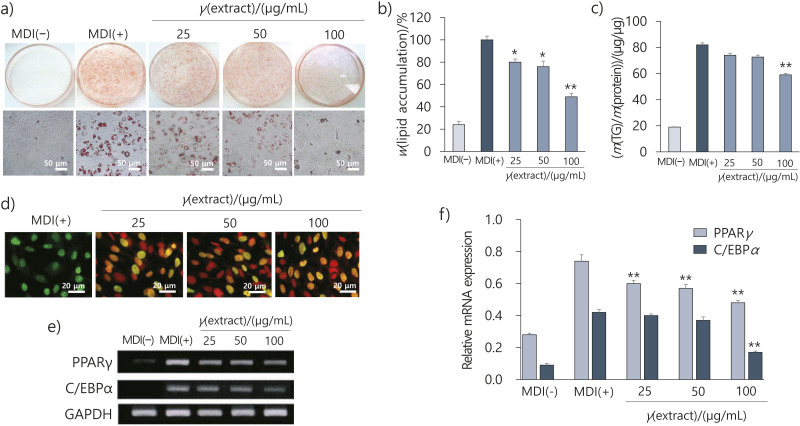
Effect of *Elsholtzia splendens* flower extract on cell proliferation and lipid accumulation during adipogenesis. Cells were differentiated into adipocytes for 8 days in a medium with or without the flower extract (25, 50 and 100 µg/mL): a) image of lipid droplets of adipocytes stained with Oil Red O (ORO), b) total lipid content of adipocytes treated with the flower extract, c) intracellular triglyceride (TG) content of adipocytes treated with the flower extract, d) cell proliferation investigated by bromodeoxyuridine (BrdU) immunostaining, e) bands indicate expressed PPARγ, C/EBPα and GAPDH mRNAs, and f) graph showing PPARγ/GAPDH and C/EBPα/GAPDH ratios. Results are shown as the mean value±S.D. of three independent experiments. The significant differences between the MDI-treated control and 3T3-L1 cells treated with the flower extract are indicated by *p<0.05 or **p<0.01. PPARγ=peroxisome proliferator-activated receptor gamma, C/EBPα=CCAAT/enhancer‐binding protein α, GAPDH=glyceraldehyde-3-phosphate dehydrogenase, MDI(+)=differentiation induction using MDI cocktail (0.5 mM isobutylmethylxanthine (IBMX), 0.5 μM dexamethasone and 10 μg/mL insulin), MDI(–)=no induction

Cells treated with the flower extract showed a marked, dose-dependent inhibition of lipid accumulation in the cytoplasm compared to the MDI-treated cells (Fig. 3a and Fig. 3b). In particular, the flower extract at 100 μg/mL resulted in a 50 % decrease in lipid accumulation compared to MDI-treated cells. To further investigate the effect of the flower extract on adipogenesis, cellular TG content in adipocytes was measured. The flower extract (100 μg/mL) significantly reduced intracellular TG content to (57.6±0.9) μg/μg (p<0.01), compared to MDI-treated cells ((82.0±1.7) μg/μg) (Fig. 3c).

To determine the effect of the flower extract on preadipocyte proliferation, we performed a BrdU/PI assay on day 8 of differentiation. As shown in Fig. 3d, the flower extract significantly inhibited proliferation.

The mRNA expression of PPARγ and C/EBPα, with or without the flower extract (10–100 μg/mL), in MDI-treated cells was measured by qPCR. In cells treated with the flower extract (100 μg/mL), the mRNA expression of PPARγ and C/EBPα was reduced by 30 and 55 % (p<0.01), respectively, compared to cells treated with MDI (Fig. 3e and Fig. 3f).

**Fig. 4:**
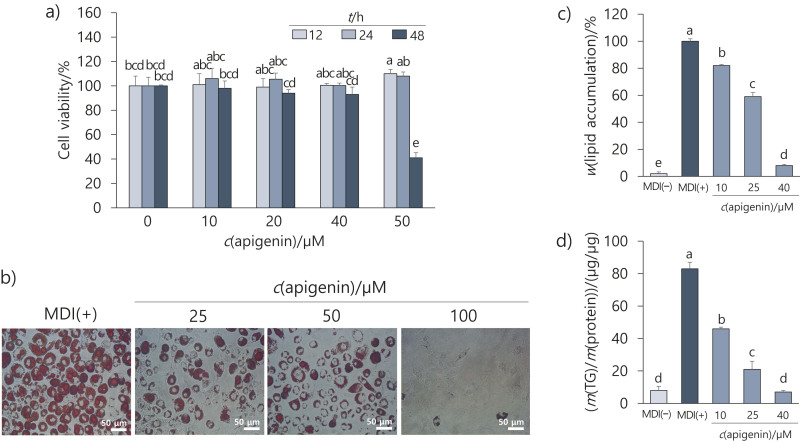
Effect of apigenin on lipid accumulation during adipogenesis. Cells were differentiated into adipocytes for 8 days in a medium with or without apigenin (10, 20 and 40 µM): a) effect of apigenin on 3T3-L1 cell viability measured by MTT assay, b) image of lipid droplets of adipocytes stained with Oil Red O (ORO), c) total lipid content of apigenin-treated adipocytes, d) intracellular triglyceride (TG) content of apigenin-treated adipocytes. Results are shown as the mean value±S.D. of three independent experiments. Different letters above bars indicate significant differences at p<0.05. MDI(+)=differentiation induction using MDI cocktail (0.5 mM isobutylmethylxanthine (IBMX), 0.5 μM dexamethasone and 10 μg/mL insulin), MDI(–)=no induction

It was necessary to confirm whether the efficacy of the flower extract was due to apigenin, its primary content. To evaluate its effect on cell viability, apigenin was administered to 3T3-L1 cells at 10–50 µM for 48 h. Cell viability decreased at 50 µM after 48 h; therefore, we used concentrations of 10-40 µM in further tests (Fig. 4a).

Apigenin inhibited lipid and TG accumulation during adipogenesis (Fig. 4b, Fig. 4c and Fig. 4d). Specifically, apigenin at 40 µM reduced lipid and TG content by 92 and 80 %, respectively, which confirmed that the anti-adipogenic effect of the flower extract on adipogenesis was caused by apigenin.

Taken together, the flower extract has been shown to inhibit lipid accumulation by regulating both proliferation and differentiation processes during adipogenesis of 3T3-L1 cells. In addition, during adipogenesis, the flower extract was found to inhibit the increased expression of PPARγ and C/EBPα transcription factors associated with differentiation. These results suggest that the effects of the flower extract might be due to apigenin. Previous studies have demonstrated that apigenin inhibits lipid accumulation by lowering the expression of PPARγ and C/EBPα during the differentiation of 3T3-L1 cells (*36*, *37*). Our conclusion strongly supports their work.

### Inhibitory effect of the flower extract and apigenin on lipid and TG accumulation in C. elegans

We used *C. elegans* as an experimental animal model to determine whether the effects of the flower extract could be observed not only at the cellular level but also *in vivo*. This inhibitory effect of the flower extract on lipid accumulation has been confirmed both at the cellular level and in animal models. Because *C. elegans* has a transparent body, individual cells are easily visualized by staining (*38*). Thus, we were able to induce obesity in *C. elegans* with a high-glucose diet and then observe the phenotypic and physiological responses.

**Fig. 5:**
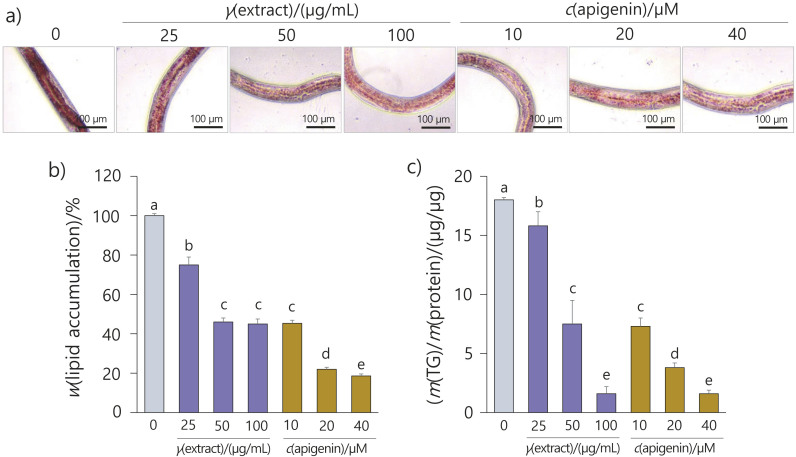
The inhibitory effect of *Elsholtzia splendens* flower extract and apigenin on lipid accumulation in *Caenorhabditis elegans*. L4 stage worms were treated with the flower extract (25, 50 or 100 µg/mL) or apigenin (10, 20 or 40 µM) for 12 h: a) images of *C. elegans* lipid droplets stained with Oil Red O (ORO), b) total lipid content of *C. elegans* treated with the flower extract or apigenin, and c) triglyceride (TG) content of *C. elegans* treated with the flower extract or apigenin. Results are shown as the mean value±S.D. of three independent experiments (*N*≥20). Different letters above bars indicate significant differences at p<0.05

As observed in 3T3-L1 cells, lipid and TG accumulation decreased in the groups treated with the flower extract and apigenin (Fig. 5). When nematodes rendered obese by a high-glucose diet were exposed to ORO solution, their deposited body fat stained strongly red, and the intensity of the red colour was reduced when the flower extract was administered (Fig. 5a). In particular, apigenin at 40 µM significantly inhibited lipid and TG accumulation by 82 and 89 % compared to the control, respectively (Fig. 5b and Fig. 5c), which showed that the effects of the flower extract and apigenin occur at the cellular level and in animal systems. Choi and Kim (*39*) reported a dose-dependent decrease in blood TG and LDL in mice fed *E. splendens* extracts. Although they did not observe morphological changes in adipose tissue, they demonstrated that *E. splendens* extracts improved lipid metabolism in mammals (*39*).

### Effect of the flower extract and apigenin on the nuclear translocation of DAF-16 in C. elegans

**Fig. 6:**
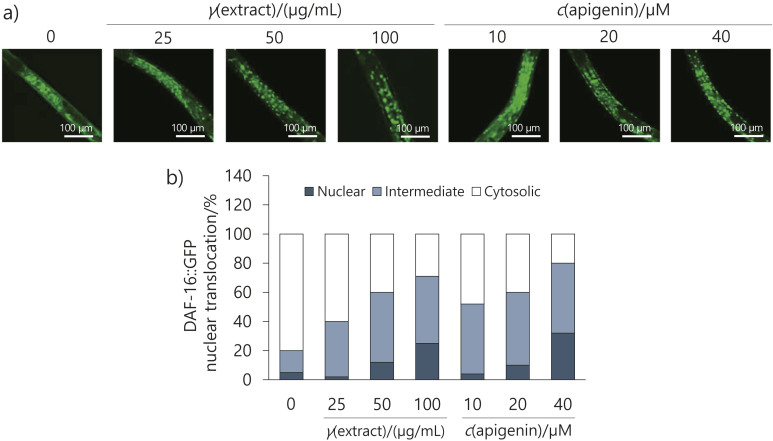
*Elsholtzia splendens* flower extract and apigenin induced the nuclear translocation of DAF-16 in *Caenorhabditis elegans*. Nematodes were treated with the flower extract (25, 50 or 100 µg/mL) or apigenin (10, 20 or 40 µM) for 72 h, then the subcellular localization of DAF-16::GFP was observed: a) representative images of the transgenic strain TJ356 worm showing cytosolic, intermediate and nuclear DAF-16 localization, b) activation of DAF-16 nuclear translocation by the flower extract and apigenin. Results are shown as the mean value±S.D. of three independent experiments (*N*≥20)

To assess whether the flower extract and apigenin respond to DAF-16/FOXO under a high-glucose diet, we used nematodes with a GFP-tagged DAF-16 protein and monitored the nuclear translocation of DAF-16. We observed an increase in the nuclear translocation of DAF-16 dependent on the concentration of the flower extract and apigenin (Fig. 6). In other words, we have demonstrated that DAF-16 is involved in the metabolic stress caused by a high-glucose diet and that the flower extract and apigenin can modulate this metabolism.

DAF-16/FOXOs are associated with insulin resistance and regulate energy metabolism (*9*, *10*). FOXO1, in particular, is involved in cell cycle regulation during adipocyte differentiation and ultimately influences cell death or proliferation (*9*-*11*). FOXO1 also regulates adipocyte differentiation by modulating the expression of PPARγ (*9*-*11*, *40*). This study strongly suggests that the flower extract relies on FOXO to inhibit lipid accumulation by: (*i*) regulating the cell cycle to inhibit preadipocyte proliferation, and (*ii*) regulating PPARγ to inhibit differentiation into adipocytes. Nevertheless, there are several limitations to this study. We did not identify a direct relationship between FOXO and cell proliferation or differentiation in either cell or nematode models at the molecular level. Therefore, further studies should elucidate the downstream molecular mechanism of FOXO in the inhibitory effect of the flower extract during adipogenesis in independent experimental models.

### Effect of the flower extract and apigenin on ROS accumulation in C. elegans

**Fig. 7:**
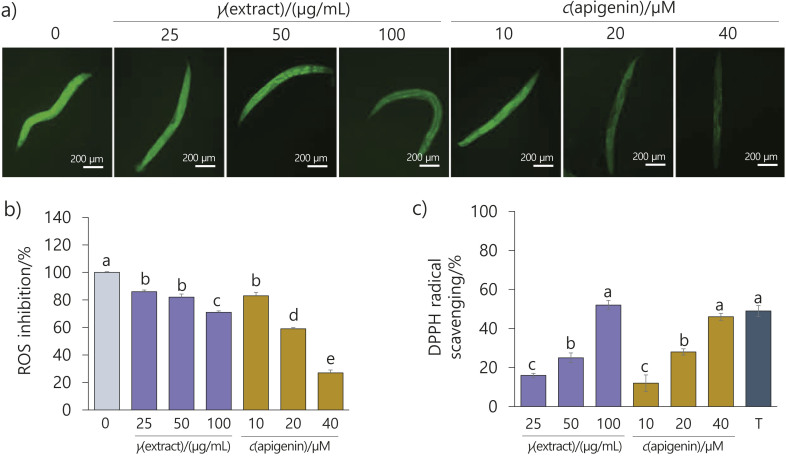
Effect of *Elsholtzia splendens* flower extracts and apigenin on reactive oxygen species (ROS) inhibition in *Caenorhabditis elegans*. L2 stage worms were treated with the flower extract (25, 50 or 100 µg/mL) or apigenin (10, 20 or 40 µM) for 50 h: a) images of green fluorescent ROS of *C. elegans*, b) quantitative ROS inhibition in *C. elegans* treated with the flower extract or apigenin (*N*≥20), and c) DPPH radical scavenging activity of the flower extract and apigenin. Results are shown as the mean value±S.D. of three independent experiments. Different letters above bars indicate significant differences at p<0.05. T=Trolox 40 μM

A previous study investigated whether glucose induces oxidative stress and found that it does in a glucose concentration-dependent manner in nematodes (*12*). Therefore, we induced oxidative metabolic stress with 2 % glucose. Nematodes treated with the flower extract and apigenin showed a concentration-dependent inhibition of the accumulation of reactive oxygen species (ROS) compared to the control (Fig. 7a and Fig. 7b). *In vitro* radical scavenging activity also showed that the flower extract at 100 µg/mL and apigenin at 40 µM had similar effects to the control, 40 µM Trolox (Fig. 7c). Several studies have also reported that the flower extract and apigenin have antioxidant properties (*20*, *24*, *25*). Meanwhile, numerous studies have demonstrated the interplay between ROS and fat accumulation in the context of metabolic diseases, with the involvement of DAF-16 (*8*, *41*). In addition, previous reports have shown that nuclear localization of DAF-16 is a prerequisite for the activation of antioxidant-related genes, including Mn-SOD (*sod-3*) and catalases (*ctl-1* and *ctl-2*), which regulate ROS production (*12*, *42*, *43*). These findings have led to the hypothesis that the flower extract might modulate FOXO translocation into the nucleus to reduce ROS, thereby ameliorating obesity and its complications, metabolic diseases. Indeed, in the present study, the intranuclear expression of DAF-16 was increased and ROS were reduced by the antioxidant properties of the flower extract and apigenin under oxidative stress conditions caused by high glucose intake in nematodes.

In obesity, enlarged fat cells lead to lipid metabolism imbalance and oxidative stress, resulting in excessive ROS production. ROS contribute to metabolic disorders, such as inflammation and insulin resistance, further exacerbating lipid imbalance and promoting obesity-related diseases (*13*). Therefore, the inhibition of lipid accumulation and ROS production represents a dual challenge in the treatment of metabolic diseases, including obesity (*44*). A growing body of evidence has highlighted the therapeutic potential of antioxidants as adjuncts in obesity prevention and treatment, suggesting that their use may significantly improve overall health outcomes.

## CONCLUSIONS

In conclusion, *Elsholtzia splendens* flower extract and its bioactive component, apigenin, were found to reduce lipid and triglyceride (TG) levels in 3T3-L1 cells and *Caenorhabditis elegans*. The results indicate that the flower extract inhibits adipogenic differentiation, involving downregulation of PPARγ and C/EBPα at the molecular level. Furthermore, increased nuclear localization of DAF-16/forkhead box O (FOXO) was observed in nematodes. Taken together, these results suggest that the flower extract may inhibit adipose accumulation by increasing FOXO and decreasing the adipogenic factors PPARγ and C/EBPα. In addition, the flower extract inhibited ROS overproduction. Therefore, the flower extract containing apigenin could have a positive effect against obesity-related metabolic stress. Previous research on *E. splendens* has mainly focused on the plant characteristics in relation to its cultivation and growth. Our findings provide new insights, as there is a lack of research on the efficacy of *E. splendens* in metabolic diseases, including obesity. However, to advance the development of *E. splendens* as a functional food for health promotion, further studies are needed to investigate its molecular efficacy, particularly the activation of PPARγ and C/EBPα in relation to DAF-16 in mammalian models. Additionally, toxicity assessments and pharmacokinetic evaluations should be conducted to ensure its safety and effectiveness.

## Appendix

**Table S1:** Primer sequences used for PCR

Gene	Primer	Sequence
PPARγ	Forward	5’-ACC ACT CGC ATT CCT TTG AC-3'
	Reverse	5’-TCA GCG GGA AGG ACT TTA TG-3’
C/EBPα	Forward	5’-ATG GAG TCG GCC GAC TTC TAC-3'
	Reverse	5’-CAG GAA CTC GTC GTT GAA GGC-3’
GAPDH	Forward	5’-AAC TTT GGC ATT GTG GAA GGG C-3’
	Reverse	5’-GAC ACA TTG GGG GTA GGA ACA C-3’
